# Does sonification of action simulation training impact corticospinal excitability and audiomotor plasticity?

**DOI:** 10.1007/s00221-021-06069-w

**Published:** 2021-03-08

**Authors:** Fabio Castro, Ladan Osman, Giovanni Di Pino, Aleksandra Vuckovic, Alexander Nowicky, Daniel Bishop

**Affiliations:** 1 Research Unit of Neurophysiology and Neuroengineering of Human-Technology Interaction (NeXTlab), Università Campus Bio-Medico Di Roma, Rome, Italy; 2 Centre for Cognitive Neuroscience, Department of Life Sciences, College of Health, Medicine and Life Sciences, Brunel University London, Uxbridge, UK; 3 Department of Life Sciences, College of Health, Medicine and Life Sciences, Brunel University London, Uxbridge, UK; 4 School of Engineering, College of Engineering and Science, James Watt Building (South) University of Glasgow, Glasgow, G12 8QQ UK; 5 Centre for Cognitive Neuroscience, Department of Clinical Sciences, College of Health, Medicine and Life Sciences, Brunel University London, Uxbridge, UK

**Keywords:** Paired associative stimulation, Sonification, Plasticity, Transcranial magnetic stimulation, Sensory augmentation, Metaplasticity

## Abstract

Sonification is a sensory augmentation strategy whereby a sound is associated with, and modulated by, movement. Evidence suggests that sonification could be a viable strategy to maximize learning and rehabilitation. Recent studies investigated sonification of action observation, reporting beneficial effects, especially in Parkinson’s disease. However, research on simulation training—a training regime based on action observation and motor imagery, in which actions are internally simulated, without physical execution—suggest that action observation alone is suboptimal, compared to the combined use of action observation and motor imagery. In this study, we explored the effects of sonified action observation and motor imagery on corticospinal excitability, as well as to evaluate the extent of practice-dependent plasticity induced by this training. Nineteen participants were recruited to complete a practice session based on combined and congruent action observation and motor imagery (AOMI) and physical imitation of the same action. Prior to the beginning, participants were randomly assigned to one of two groups, one group (nine participants) completed the practice block with sonified AOMI, while the other group (ten participants) completed the practice without extrinsic auditory information and served as control group. To investigate practice-induced plasticity, participants completed two auditory paired associative stimulation (aPAS) protocols, one completed after the practice block, and another one completed alone, without additional interventions, at least 7 days before the practice. After the practice block, both groups significantly increased their corticospinal excitability, but sonification did not exert additional benefits, compared to non-sonified conditions. In addition, aPAS significantly increased corticospinal excitability when completed alone, but when it was primed by a practice block, no modulatory effects on corticospinal excitability were found. It is possible that sonification of combined action observation and motor imagery may not be a useful strategy to improve corticospinal, but further studies are needed to explore its relationship with performance improvements. We also confirm the neuromodulatory effect of aPAS, but its interaction with audiomotor practice remain unclear.

**Supplementary Information:**

The online version contains supplementary material available at 10.1007/s00221-021-06069-w.

## Introduction

Motor skill learning is a fundamental aspect of everyday life and injury recovery. Recent evidence exploring strategies to maximize learning suggest that action simulation training based on action observation (AO) and motor imagery (MI) could be an effective addition to the learning process (Ruffino et al. 2017; Ste-Marie et al. 2012), and rehabilitation (for a review see Abbruzzese et al. 2015; Mulder 2007). Neurophysiological and neuroimaging studies on AO and MI report activation of a similar fronto-parietal network to the one active during the physical execution of an action (PE; Filimon et al. 2007; Hardwick et al. 2018; Simos et al. 2017). In addition, investigations on the specificity of this neural activation using transcranial magnetic stimulation (TMS) report that during AO and MI, corticospinal excitability exhibits spatial, temporal and contextual similarities to PE (Grospretre et al. 2016; Naish et al. 2014). Recent investigations explored the combined effects of performing MI during AO, suggesting that combined action observation and motor imagery (AOMI) engages the brain in a more extended way, compared to the two activities alone. Neuroimaging studies report that, although AOMI engages largely overlapping networks also involved in AO and MI alone, it also exhibits distinctive neural signatures (Taube et al. 2015). Furthermore, studies using electroencephalography (EEG) showed that this increased activity is focused in alpha and beta frequency bands, which have also been involved in sensorimotor computations (Berends et al. 2013; Eaves et al. 2016a, b). The extended brain activity during AOMI is also reflected in an increased corticospinal excitability (Mouthon et al. 2015; Sakamoto et al. 2009; Wright et al. 2018). Evidence also shows that AOMI may enhance learning and performance compared with AO or MI alone, in sport contexts (Aoyama et al. 2020; Romano-Smith et al. 2018), rehabilitation, such as stroke (Sun et al. 2016) and Developmental Coordination Disorder (DCD; Marshall et al. 2020; Scott et al. 2019). The beneficial effect of combining AO and MI may be due to the complementarity of AO and MI. Indeed, AO engages visual as well as motor areas, to map the observed action into the observer’s own motor system (Friston et al. 2011), while MI entails similar processes involved in physical execution of the action (Hardwick et al. 2018), including its sensory afferences (Kilteni et al. 2018). To account for this, in recent years, a ‘dual simulation hypothesis’ has been proposed, which suggests that sensorimotor representations evoked by AO and MI may be computed simultaneously and, according to their content and perspective, could either complement or compete for neural substrates underlying sensorimotor computation (Eaves et al. 2016b; Vogt et al. 2013.

In the present study, we aimed at investigating whether it is possible to maximise the effect of AOMI on corticospinal activation with the use of sonification, an auditory augmentation strategy whereby an extrinsic sound is associated to—and modulated by—movement (Schaffert et al. 2019). The rationale for the combined use of sonification in conjunction with AOMI is that multisensory interaction improves perception and integration of sensory information into internal models of the body and the environment (D’Alonzo et al. 2020; Friston et al. 2010; Shams and Seitz 2008), thus enabling and improving the ability of the former to interact with the latter (Wolpert and Flanagan 2016). Sonification was suggested to be a viable strategy to improve performance and maximise learning (Dyer et al. 2015). Recent works on sonification explored its use in observational learning. Schmitz et al. (2013) asked participants to observe videos of a human-like avatar performing a breaststroke, where the relative distance between the wrists and ankles were associated with synthetized sounds. When the sound was congruent with the observed action, sonified action observation (sAO) yielded better perceptual judgment about movement speed, compared to incongruent conditions, where the matching between sound and action was not coherent and synchronised. In addition, congruent sonification activated areas known for their involvement in multisensory integration, such as the superior temporal sulcus and the insula, and it enhanced the strength of their connection with sensorimotor areas. More recently, Mezzarobba et al. (2018) reported that sAO of different actions, followed by physical execution of the same actions, significantly reduced freezing of gaze in people with Parkinson’s disease. So far, evidence is in favour of an additive learning effect of MI on AO, and of sonification on AO. However, it is currently unknown whether motor imagery could have an incremental effect on sAO. Recent evidence suggests that during MI, the brain simulates also the sensory consequences of the imagined movement (Kilteni et al. 2018), and a copy of the motor command (efference copy) is treated as a sensory afference and integrated with others sensory modalities (Pinardi et al. 2020). Thus, it is conceivable that the spatiotemporal information about an action obtained during AO and sonification, along with the simulated one during MI, would all converge to a better integration of a multisensory internal models. This would be in line with the dual simulation hypothesis, suggesting that congruent sensorimotor representations would facilitate the simulation of the action, and potentially afford plasticity (Eaves et al. 2016b). Thus, the first aim of this study was to investigate whether sonified AOMI (sAOMI) of a right-hand battery pinching would enhance motor cortex excitability. To investigate it, we compared practice-related changes in peak-to-peak amplitude of the motor-evoked potentials (MEPs) in two groups of participants undergoing a practice block based on AOMI. For one group, AOMI was enriched with sonification (SON group) and the other without extrinsic auditory information (CON group).

A secondary aim of this study was to gain information about audiomotor plasticity arising from the interaction between sonification, action observation and motor imagery (sAOMI) practice. To do so, we took advantage of the inter-dependency between practice and neuroplasticity, i.e., the propensity of the nervous system to change its structure and function with experience (Di Pino et al. 2014b). Studies on neural mechanisms of practice-dependent plasticity report that motor skill learning is associated with a long-term potentiation (LTP) of synaptic strength and weight within the network targeted by the learning process (Dayan and Cohen 2011; Rioult-Pedotti et al. 1998; Ziemann et al. 2001). The interaction of training and LTP-like plasticity can also be studied in humans using non-invasive brain stimulation (Cirillo et al. 2016). One such protocol is Paired Associative Stimulation (PAS) which is based on a repeated association between a sensory stimulus and a TMS pulse, inducing a long-lasting increase in corticospinal excitability (Carson and Kennedy 2013; Stefan et al. 2000; Suppa et al. 2017). This protocol can be harnessed to investigate the interaction between practice and plasticity. Evidence shows that practice interferes with the induction of the long-lasting increase in corticospinal excitability induced by PAS when done alone; That is, when practice precedes PAS, the induction of LTP-like plasticity is occluded (Di Pino et al. 2014b; Rosenkranz et al. 2007a, b; Stefan et al. 2006; Ziemann et al. 2004). Interestingly, recent evidence suggests that plasticity in the audiomotor pathway can be studied using PAS. Sowman et al. (2014) reported that pairing the utterance of a word 'Hey' with TMS stimulation delivered 100 ms after the sound onset at the FDI muscle increased corticospinal excitability by 40% immediately after the auditory PAS (aPAS) and 65% increase 15 min from its end. In this study, we employed aPAS to study the temporal interaction between a sAOMI practice and LTP-like plasticity of the audiomotor pathway artificially induced by a non-invasive neuromodulatory protocol. To do so, we administered aPAS to our participants after a practice session based on AOMI and compared the induced changes of motor cortex excitability with the ones induced in the same subject by aPAS alone performed on a different day.

## Methods

### Participants

Twenty-two self-reported neurologically and psychiatrically healthy right-handed young adults (Table 1; eight females; age: M 25.67, SE 2.08) were recruited for this study. None of them reported completing any formal musical training. Participants completed the Edinburgh Handedness Inventory to assess their hand dominance (EHI; Oldfield 1971). In addition, participants completed a TMS safety screening questionnaire (Rossi et al. 2009, 2011). Finally, participants’ vividness of MI was assessed using the Motor Imagery Questionnaire 3 (MIQ-3; Williams et al. 2012). Two participants dropped out after the first session. In addition, one more participant’s data were discarded due to compromised M-wave recording. Those participants were excluded, leaving nineteen participants to be included in the analysis. Nine participants were assigned to the SON group, and the remaining ten were assigned to the CON group. The study was approved by the Brunel University London College of Health, Medicine and Life Sciences Research Ethics Committee and data collection was in accordance with the principles of the Declaration of Helsinki.

**Table 1:** Demographic Data, by Group

	SON		CON	
	Mean	SEM	Mean	SEM
Age (years)	25.67	2.08	25.27	2.01
EHI Score	9.57	0.74	7.71	0.74
Body Weight (kg)	74.78	5.76	66.73	3.26
Body Height (cm)	171.56	3.93	172.55	2.32
Internal visual imagery	5.39	0.41	5.73	0.36
External Visual Imagery	5.97	0.29	5.64	0.32
Kinesthetic Imagery	5.11	0.48	5.45	0.44

### Experimental design

**Fig. 1:**
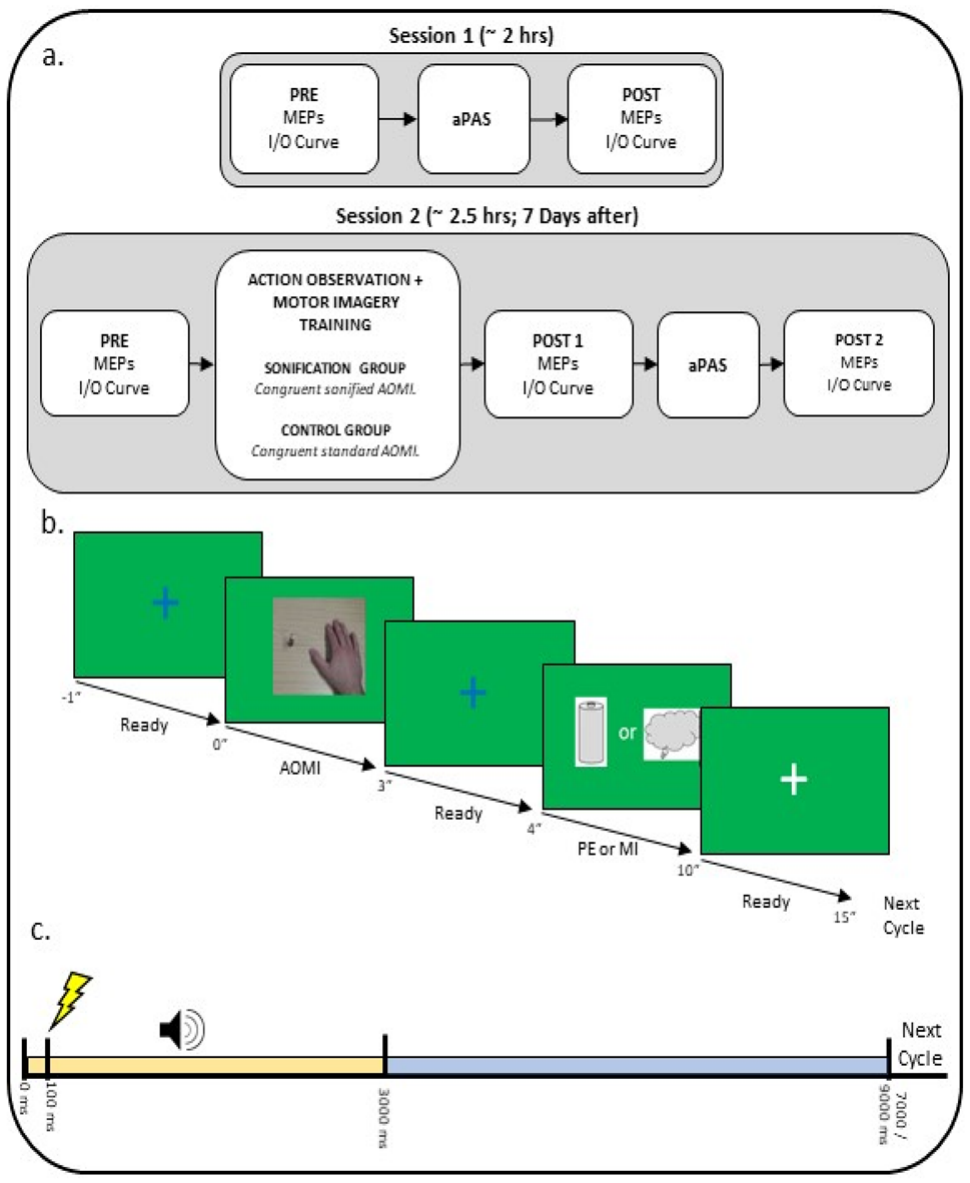
Schematic representation of the experimental design. **a** Participants visited the laboratory on two non-consecutive sessions. Session 1 was designed to investigate the effect of aPAS on corticospinal excitability. On the second session, participants engaged in a single practice block, followed by another aPAS protocol, to investigate the interaction between practice and PAS. Measures of corticospinal excitability on the first visit were obtained before and after the completion of the aPAS protocol. For the second visit, corticospinal excitability was measured at three timepoints: before the training (PRE), after the training (POST1) and after the aPAS (POST2). **b** Schematic representation of the practice session. Participants first observed a blue cross, representing a ‘ready’ cue, then engaged in AOMI; the SON group heard the sonification sound concurrently. After this, another blue cross appeared, after which participants either imagined (MI; cloud icon) or executed (PE; battery icon) the same action. When a white cross appeared, participants did nothing for a 5 s period. **c** Details of the aPAS protocol. For each audiomotor training, the TMS pulse was delivered 100 ms after the onset of the sound. The sound lasted for 3 s (yellow box). The audiomotor training was delivered every 4–6 s (blue box)

Figure 1a provides a chronological representation of the experimental design. The experiment consisted of two sessions, completed in fixed order on 2 separate days. The second session was completed after at least 7 days, to prevent carryover influences of the aPAS on the first session (Ziemann et al. 2004). In the first session, participants completed an aPAS protocol alone. This session served as a baseline for comparison with data from the second session. Corticospinal excitability was assessed before (PRE) and after (POST) the aPAS protocol. The second session was designed to assess the influence of sonification on corticospinal excitability, and audiomotor plasticity arising from the training. Participants completed a practice block composed of congruent AOMI followed by either MI or PE of the same action (see later for more details). In this practice session, participants were randomly assigned to two groups: SON group engaged in sAOMI, while CON completed the session without extrinsic auditory information. After the practice, participants completed another aPAS protocol, which allowed us to investigate the audiomotor-induced plasticity arising from the training. In the second session, corticospinal excitability was measured at three time points: Before (PRE) and after (POST 1) the practice block, and after the aPAS session (POST 2).

### Combined action observation and motor imagery practice

During the second experimental session, both groups completed a single AOMI practice block, comprising 96 trials for a total duration of approximately 30 min. Trials were split into six blocks, with a 1-minute break in between the blocks, to allow the participant to relax. Figure 1b depicts a schematic representation of the stimuli presentation during each trial. Participants first observed the action and were asked to concurrently imagine executing the same action using kinesthetic motor imagery. During AOMI, SON group listened to the sonification sound, while CON group did not hear any extrinsic sound. After that, a blue cross appeared for 1 s, notifying them to get ready, after which an icon indicated that they should either imagine (thought bubble icon) or imitate (battery icon) the action. After this, participants pushed the ‘enter’ button on a numeric keypad, to terminate the trial, and rest for 5 s, after which a new trial begun, by showing another blue cross. Ten trials in each block required the participants to engage in MI; the remaining ones required them to perform the action (PE). We chose to add physical execution trials, because we sought to design a practice protocol that was as similar as possible to one that would be carried out in applied settings. It has been argued that, although it is possible to learn an action using just MI (Kraeutner et al. 2015), physical execution of an action remains a fundamental component in motor learning (Mulder et al. 2004). Previous research has highlighted the benefits of execution trials in mental practice (Ruffino et al. 2017), and evidence from clinical studies show that people that who cannot execute movements, such as in spinal cord injury, can attempt at perform it, with beneficial effects for performance (Mateo et al. 2015) MI and PE trials were fully randomized; PE occurring 25% of trials.

### Task and sonification process

Participants observed an actor pinching a battery with his right thumb and index finger, an action that was either sonified (SON) or not (CON). Sonification was performed using a frame-by-frame strategy. Raw videos were recorded using a Sony HDR-TD30, and images were acquired at 25 frames per second, at a resolution of 1920 × 1080 pixels. The raw files were exported in the free video editing software *Hitfilm express 2017* (FXHOME Limited, UK) for sonification. We chose to sonify the distance between the thumb and the index finger. The sonification sound chosen was a synthesized pitch, which was created in the opensource software *Audacity*. The sound was first created and then matched with the video in *Hitfilm express 2017* (FXHOME Limited, UK). We chose a synthetized sound because we were interested in exploring the potential use of non-action sound, auditory stimuli that do not evoke audiomotor resonance per se. Research shows that these sounds can be effectively associated to the representation of an action (Ticini et al. 2011). In addition, our audiomotor mapping is the most used in sonification research, as per a recent systematic review (Dubus and Bresin 2013).

### Assessment of corticospinal excitability

To measure changes in corticospinal excitability as a result of the intervention and aPAS, we investigated changes in peak-to-peak amplitude MEPs of the right first dorsal interosseous (FDI) muscle, a muscle that was involved in the action. TMS pulses were delivered at 130% of individual’s resting motor threshold (rMT). In addition, we investigated the input–output relationship of MEPs (IO curve). For this test, MEPs were collected at the intensities of 80%, 90%, 100% (rMT), 105%, 110%, 120%, 130%, 140% and 150% of rMT. A total of 90 pulses were randomly delivered, 10 per stimulation intensity. The IO curve assesses differential recruitment of different motor units with increasing stimulation intensity (Carroll et al. 2001; Devanne, Lavoie, & Capaday, 1997). Both MEP and IO curve data were collected because the latter may be necessary when the protocol implies measuring corticospinal excitability across multiple days, as is more robust to possible confounds, such as intertrial changes in coil position and orientation (Rossini et al. 2015).

Participants sat on a chair in front of a 24″ LCD monitor (model XL2430-B, BENQ) at a viewing distance of one meter. They were instructed to position their arms and elbows on the table, keeping their hands in a pronated and relaxed position. Muscle activity was monitored throughout the experiment. Participants were continuously reminded to relax as much as possible, and not to move during the stimulation periods. TMS responses were delivered using a Magstim 200 delivering monophasic pulses (Magstim Company, Whitland, UK), using a 70 mm figure-of-eight stimulation coil, oriented as to induce posterior-to-anterior current. MEPs were collected using Ag/AgCl electrodes (Kendall, Covidien, Canada) arranged in a bipolar, belly-tendon montage. To reduce skin resistance, participants’ skin area was shaved (if necessary), abraded using an abrasive paste and cleaned using isopropyl alcohol swabs. After the preparation of the participant, the hotspot for TMS stimulation was found. Hotspot identification began by placing the coil 5 cm lateral and 1 cm anterior to the individually defined apex. From this position, the hotspot was defined as the coil position and orientation that evoked MEPs of the largest amplitude at the same stimulation intensity. The position was marked on the scalp with a soft-tip pen, to allow repositioning of the TMS coil after the breaks. Subsequently, the resting motor threshold (rMT) was determined using adaptive threshold hunting technique (Ah Sen et al. 2017; Awiszus 2011). This allowed us to determine the rMT with a reduced number of TMS pulses, thereby improving participants’ comfort, and reducing total testing time. During all periods of TMS stimulation, participants were asked to direct their visual attention to a fixation cross at the center of a screen and to count down from 200 to 0 (Kumpulainen et al. 2014). At the end of each session, FDI M-waves were collected to normalize MEPs across participants. This was done using peripheral magnetic stimulation of the ulnar nerve, which was obtained by placing the TMS coil on the elbow, between the olecranon and the medial epicondyle, with the coil handle perpendicular to the direction of the ulnar nerve, to induce current flow in the nerve with the monophasic stimulator (Lampropoulou et al. 2012). To determine M-max, we collected five evoked M-waves responses from intensities ranging from 20 to 70% of the maximum stimulator output, with incremental steps of 10%. Surface electromyography and evoked responses were recorded using Signal (v. 6, CED, UK) and amplified at a gain of 1000 and sampled at 4 kHz. To reduce the influence of external artefacts, an online band-pass filter (5–2000 Hz) was applied. TMS was applied through synchronized stimulus presentation, using TTL output triggers generated by E-Prime software (v 3.0; Psychology Software Tools, Pittsburgh, PA), and sent to the magnetic stimulator.

### Auditory paired-associative stimulation (aPAS)

The aPAS protocol (Fig. 1c) consisted of 200 audiomotor pairings, each of which consisted of an auditory stimulus and a TMS pulse. The protocol was controlled using E-Prime, which was used to time the TMS pulse in relation to the auditory stimulus. The pairing auditory stimulus was a pre-recorded sound of fingers typing on a computer keyboard, and the TMS pulse was delivered 100 ms after the sound onset, with stimulus intensity set at 120% rMT. The auditory stimulus was played for 3000 ms. The interstimulus interval (ISI) between sound onset and TMS pulse was chosen in accordance with previous research on aPAS (Sowman et al. 2014). The pairs of stimuli were delivered with a random interval between 4000 and 6000 ms. The pairings were organized in 4 blocks of 50 pairings each, with one minute of rest between blocks. Auditory stimulation was delivered via in-ear earphones. Sound volume was adjusted for each participant so that it was comfortable to hear the sound, without perceived distortions. During the protocol, participants were asked to direct their gaze to a white fixation cross on the screen, and to pay attention to the sound. Prior to the beginning of the protocol, the sound was played, and all participants successfully reported to recognize the action sound.

### Data and statistical analysis

*MEPs Analysis* All data were stored on an external drive for offline analysis. For each trial, MEPs peak-to-peak amplitude and background EMG levels were calculated using a custom-made script in Signal software (CED, v 6.05; UK), and then exported to Microsoft Excel for further analysis. Muscle activity prior to the TMS pulse was calculated as a root mean square of background EMG during the 100 ms prior to the TMS pulse. Trials with background EMG levels greater than 300 µV were excluded from MEPs analysis. With this threshold, less than 1% of the total number of MEPs were removed from the analysis. Raw MEPs were normalized and expressed as a percentage of the maximal evoked muscle response (*M*_max_), obtained for each participant at the end of each testing session, using the following formula (henceforth, MEPs will refer to normalized, not raw, MEPs):

1 $$\text{Normalised MEP}=100\times \frac{\mathrm{MEP}}{M_\text{max}}.$$

We chose this normalization method because *M*_max_ is thought to thought to be stable across time, as it represents the maximal activation of the α motoneuron pool, in this case evoked by peripheral magnetic stimulation (Lampropoulou et al. 2012; Palmieri 2004). Thus, this gave a stable comparison for MEPs, which are influenced by different activities (Bestmann et al. 2015; Klein-flu et al. 2013).

*IO Curve Analysis*

The relationship between TMS stimulation and MEP response, was investigated by fitting a four parameter Boltzmann sigmoid function over the MEPs of the nine stimulation intensities. Peak-to-peak amplitude and bgEMG was calculated using the same script. We averaged MEPs for each stimulation intensity. Curve fitting was performed using the built-in sigmoid curve fitting features of Signal software (CED, v 6.05, UK). The fitting was done using the following equation:

2 $$\mathrm{MEP}\left(I\right)=\frac{{\text{MEP}}_{\text{max}}-{\text{MEP}}_{\text{min}}}{1+e\frac{\mathrm{I}50-1}{s}},$$

where MEP_max_ and MEP_min_ are the maximum and minimum asymptote, respectively; *I*_50_ is the stimulus intensity needed to evoke MEPs that are 50% of MEP_max_, and *s* is the slope of the curve. Curve fitting with Boltzmann equation provided several parameters, which were then used to characterize changes in corticospinal excitability as a result of protocol intervention (Carroll et al. 2001; Devanne et al. 1997). In addition to the parameter in the equation above, another index was calculated, slope I_50_, which represented the slope of the ascending phase of the curve at I_50_, which was calculated according to the following formula:

3 $$\mathrm{Slope} \, I50= \frac{m\times \mathrm{MEP}_\text{max}}{4},$$

where *m* is the slope parameter of the Boltzmann sigmoid function.

*Statistical Analysis* Statistical analysis was carried out in SPSS. Outliers in the data were assessed using z scores. Values greater than ± 2.99 were considered outliers and discarded from the analysis. Data distribution was assessed via the Shapiro–Wilk test. A paired-sample *t* test was used to assess statistical differences in rMT between sessions, while an independent *t* test was used to assess group difference in rMT in the second visit. The same tests were also used to assess between groups differences in vividness of motor imagery, by analyzing the three output of the MIQ questionnaire, internal visual imagery (IVI), external visual imagery (EVI) and kinesthetic motor imagery (KI). Lastly, between-days changes in *M*_max_ were calculated using a paired-sample t-test. Some of the indices were not normally distributed (*p* > 0.05), so non-parametric statistical analyses were used instead. Homogeneity of variance was assessed using Levene's test for equality of variances. To assess the effects of aPAS alone (on experimental session 1), we performed non parametric test on pre- and post-aPAS MEPs. Wilcoxon signed-rank test was used to assess statistical differences on IO curve indices. To assess the effects of the practice block on corticospinal excitability, and its priming effect for aPAS, we performed a mixed ANOVA with factors TIME and GROUP. TIME factor had three levels—pre-training, post-training (post 1) and post-aPAS (post 2), and GROUP two (SON and CON). In addition, we also analyzed the percentage change of corticospinal excitability after the two sessions. To this end, we performed a mixed ANOVA with factors ‘TIME’ (two levels:’aPAS D1′ and’aPAS D2′) and GROUP (two levels, SON and CON). For IO curve indices, six parameters were analyzed, MEPmax, MEPmin, MEP range, slope, I50 and slope I50. For each of these indices, an individual Sphericity of covariance was assessed with Mauchly's test of sphericity. In case of violation of sphericity, Greenhouse–Geisser epsilon adjustment was used. Bonferroni correction was applied for post hoc comparisons.

## Results

There were no significant differences in rMT between the first (38 ± 5%) and the second visit (38 ± 4%), *t*(19) = 0.151, *p* = 0.882. In addition, there were no statistically significant differences in rMT between the SON (37 ± 4%) and CON group (38 ± 4%) on the second visit, *t*(17) = − 0.612, *p* = 0.55. The MIQ-3 analysis showed no significant differences between the groups in self-reported vividness of Internal Visual Imagery (*t*(19) = − 0.49, *p* = 0.63), External Visual Imagery (*t*(19) = 0,62, *p* = 0.54), or Kinaesthetic Imagery (*t*(19) = − 0.36, *p* = 0.72). No statistically significant differences were found in Mmax between the first (11.38 ± 4.22 mV) and the second (11.70 ± 4.52 mV) visits, as assessed using a Wilcoxon signed-rank test (*z* = − 0.181, *p* = 0.856).

### Session 1: Effects of aPAS on corticospinal excitability

**Fig. 2:**
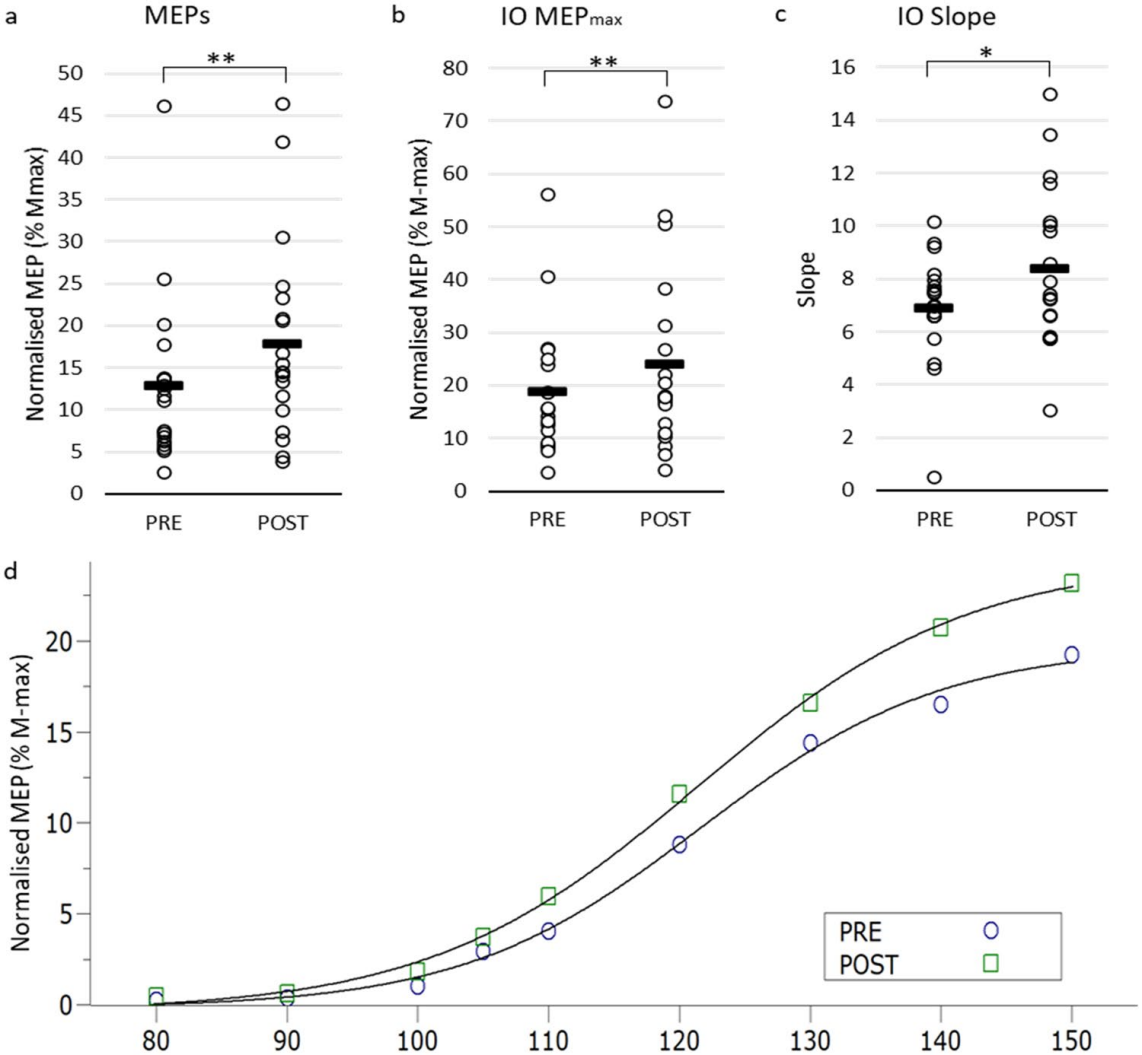
Corticospinal excitability measures before and after aPAS. On the first visit, both SON and CON completed the same protocol, so the data shown represent the group average of both groups (*n* = 19) **a** MEPs collected at 130% rMT, **b** MEPmax of the IO curve sigmoid fitting; **c** slope of the IO curve sigmoid fitting; **d** sigmoid fitting of the nine IO curve stimulation intensities for pre- and post-aPAS. White circles represent individual data, while black rectangles represent group means. *: *p* < 0.05; **: *p* < 0.01

Figure 2 and Table 2 provide a summary of the results for the first session. The aPAS protocol induced a significant increase in peak-to-peak MEP size (Fig. 2a), as compared with pre-aPAS measure: z = 3.058, *p* = 0.002). Figure 2d reports the IO curve fitting with Boltzmann function. Analysis on the indices arising from curve fitting reported a significant increase in MEP_max_ (z = 2.495, p = 0.013; Fig. 2b), slope of the fitted curve (z = 2.012, *p* = 0.44, Fig. 2c), and range of MEP responses (z = 2.535, *p* = 0.11). No significant differences were found for MEPmin (*p* = 0.136), I_50_ (p = 0.390), and the slope at I_50_ (*p* = 0.601).

**Table 2:** Descriptive Statistics for Corticospinal Excitability Measures – Visit 1 [All participants; *n* = 19]. MEPs were normalised as percentage of *M*_max_

		Mean	Median	SD	SEM	95% confidence interval	
						Lower	Upper
*MEPs at 130% rMT*							
MEP	Pre	12.89	11.63	9.89	2.27	8.13	17.67
	Post	17.9	14.45	11.63	2.66	12.3	23.5
*IO Curve*							
MEP_min_	Pre	0.15	0.10	0.39	0.09	− 0.04	0.34
	Post	− 0.20	− 0.12	0.90	0.21	− 0.63	0.23
MEP_max_	Pre	18.76	15.53	12.67	2.91	12.65	24.86
	Post	23.94	17.68	18.06	4.14	15.23	32.64
*I*_50_	Pre	120.03	119.38	6.02	1.38	117.13	122.94
	Post	118.69	117.95	8.12	1.86	114.78	122.60
Slope	Pre	6.89	6.98	2.10	0.48	5.88	7.90
	Post	8.39	7.41	3.03	0.70	6.93	9.85
MEP Range	Pre	18.61	15.17	12.75	2.93	12.46	24.76
	Post	24.14	18.29	18.71	4.29	15.12	33.16
Slope I_50_	Pre	1.05	0.51	1.75	0.40	0.20	1.89
	Post	0.74	0.45	0.52	0.12	0.49	0.99

### Session 2: Effects of AOMI training practice on corticospinal excitability and practice-dependent plasticity

**Fig. 3:**
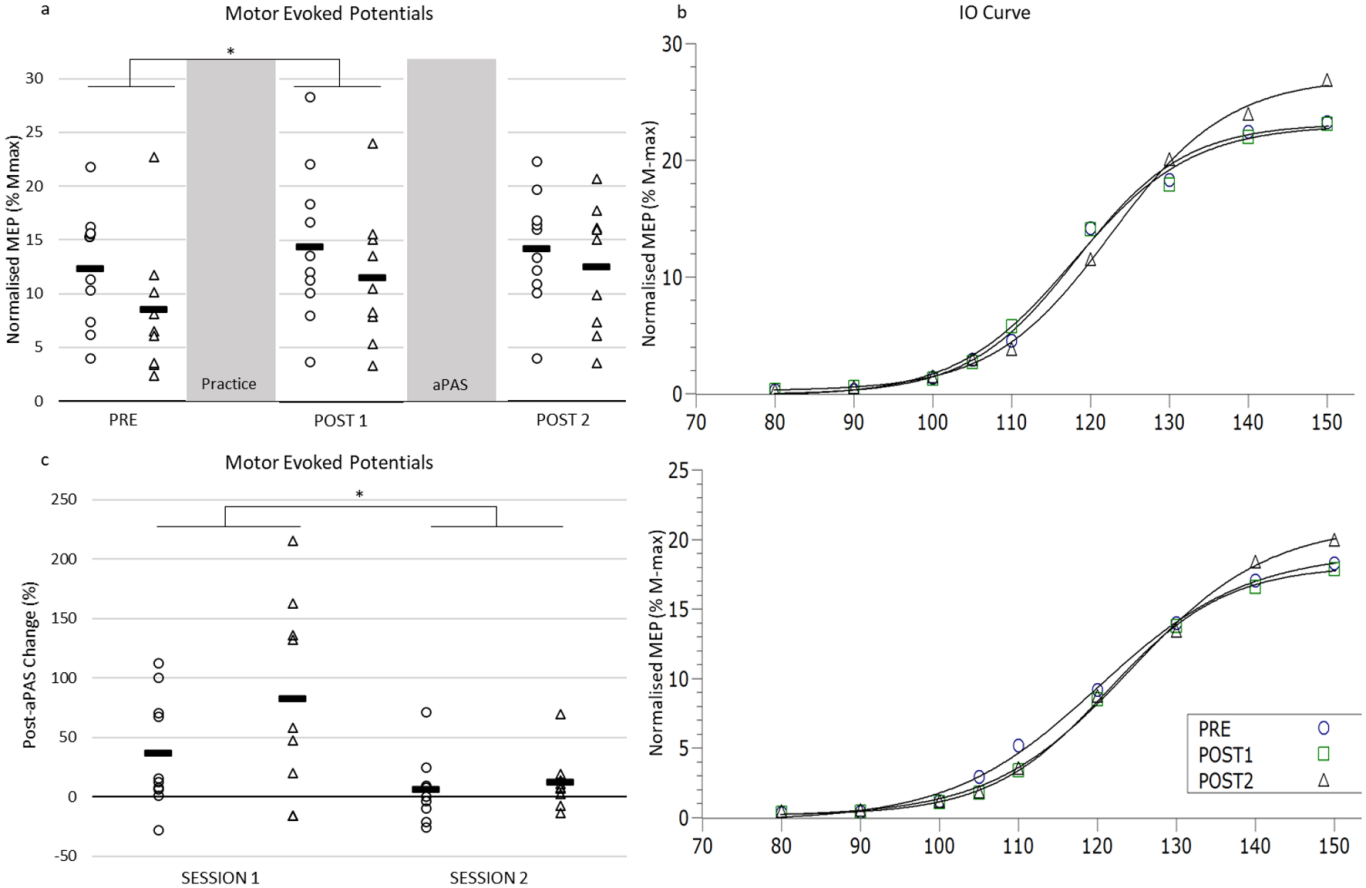
Second visit. MEPs were collected at three time points: Before the practice session (PRE), after the practice session (POST 1), and after the aPAS (POST 2). **a** Corticospinal excitability on the second visit for SON and CON groups. **b** Sigmoid fitting of the 9 IO curve stimulation intensities for PRE and POST 1 and POST2 for CON group (upper panel) and SON group (lower panel). **c** Between-days effects of aPAS on corticospinal excitability. In session 1, aPAS was the only intervention, while on session 2, aPAS was administered after the practice block. Circles and triangles represent individual values for SON and CON group, respectively. Black rectangles represent group means. *p < 0.05

Figure 3 and Table 3 provide a summary of the main results for the second session. There was a main effect of ‘TIME’ on peak-to-peak MEP amplitude: *F*(2,34) = 7.397, *p* = 0.002, η2_p_ = 0.303. No interaction TIME x GROUP on peak-to-peak MEP amplitude was found: *F*(2,34) = 0.972, *p* = 0.389, η2_p_ = 0.054. Post hoc analysis using Bonferroni correction revealed that MEP mean amplitude significantly increased after the training, as compared with pre-training values (*p* = 0.015, Fig. 3a). No significant changes were found between POST1 and POST2, suggesting that post-training aPAS did not significantly change corticospinal excitability. For the analysis of the parameters IO curve (Fig. 3b) arising from the Boltzmann fitting, a mixed ANOVA reported no main effects of TIME, nor TIME x GROUP interaction for any parameter (details on statistical analysis reported in table S1, supplementary material).

**Table 3:** Descriptive Statistics for Corticospinal Excitability Measures – Visit 1 [CON group *n* = 10; SON group n = 9)]. MEPs were normalised as percentage of M_max_

		Mean	Median	SD	SEM	95% CI	
						Lower	Upper
*SON Group*							
MEPs at 130% rMT							
MEP	Pre	12.34	13.3	5.51	1.74	8.41	16.28
	Post 1	14.35	12.79	7.11	2.25	9.26	19.42
	Post 2	14.18	14.68	5.26	1.66	10.4	17.92
IO curve							
MEP_min_	Pre	− 0.11	− 0.03	0.83	0.28	− 0.74	0.53
	Post 1	0.33	0.23	0.66	0.22	− 0.18	0.83
	Post 2	0.31	0.40	1.11	0.37	− 0.54	1.16
MEP_max_	Pre	19.37	19.44	7.27	2.42	13.79	24.96
	Post 1	18.01	16.80	6.86	2.29	12.74	23.28
	Post 2	20.03	14.34	8.70	2.90	13.34	26.71
I_50_	Pre	118.38	119.52	4.65	1.55	114.81	121.96
	Post 1	120.46	118.85	3.54	1.18	117.74	123.18
	Post 2	120.11	119.19	5.46	1.82	115.91	124.31
Slope	Pre	7.82	7.85	1.93	0.64	6.33	9.30
	Post 1	6.62	6.91	1.58	0.53	5.41	7.83
	Post 2	6.06	6.05	2.51	0.84	4.13	7.99
MEP Range	Pre	19.48	18.73	7.66	2.55	13.59	25.37
	Post 1	17.68	16.44	6.74	2.25	12.51	22.86
	Post 2	19.72	14.46	8.78	2.93	12.97	26.47
Slope I_50_	Pre	0.64	0.70	0.21	0.07	0.48	0.80
	Post 1	0.72	0.69	0.35	0.12	0.45	0.99
	Post 2	0.97	0.70	0.61	0.20	0.50	1.44
*CON Group*							
MEPs at 130% rMT							
MEP	Pre	8.29	6.51	6.26	2.09	3.48	13.10
	Post 1	11.50	10.49	6.29	2.10	6.66	16.34
	Post 2	12.50	15.03	5.92	1.97	7.95	17.05
IO curve							
MEP_min_	Pre	− 0.01	0.17	0.67	0.21	− 0.49	0.47
	Post 1	− 0.10	0.10	1.00	0.32	− 0.82	0.61
	Post 2	0.50	0.46	0.64	0.20	0.05	0.95
MEP_max_	Pre	17.12	13.90	12.67	4.01	8.05	26.18
	Post 1	17.51	15.32	11.21	3.54	9.49	25.53
	Post 2	20.50	18.79	12.95	4.09	11.24	29.77
* I*_50_	Pre	121.08	119.68	5.11	1.62	117.42	124.74
	Post 1	119.86	119.52	7.11	2.25	114.77	124.95
	Post 2	122.89	122.00	8.41	2.66	116.87	128.91
Slope	Pre	9.20	9.08	3.02	0.96	0.64	0.07
	Post 1	8.02	7.25	2.65	0.84	6.12	9.91
	Post 2	6.95	8.29	3.11	0.98	4.73	9.17
MEP Range	Pre	17.13	13.52	13.03	4.12	7.81	26.45
	Post 1	17.62	15.37	11.40	3.61	9.46	25.77
	Post 2	20.00	18.13	12.99	4.11	10.71	29.29
Slope I_50_	Pre	0.53	0.38	0.47	0.15	0.19	0.87
	Post 1	0.58	0.50	0.43	0.14	0.28	0.89
	Post 2	1.82	0.65	3.76	1.19	− 0.87	4.50

### Between-days effects of aPAS

Both groups showed a decrease in aPAS effect on the second visit, compared to the first one, expressed as a post-aPAS percentage change in MEP peak-to-peak amplitude (Fig. 3c, Table 4). A mixed ANOVA revealed a main effect of TIME on percentage change of MEP peak-to-peak amplitude following the aPAS on the two experimental sessions: *F*(1,17) = 8.183, *p* = 0.011; η2_p_ = 0.325. No interactions TIME x GROUP were found: *F*(1,17) = 1.275, *p* = 0.274; η2_p_ = 0.07.

**Table 4:** Descriptive statistics for the effect of aPAS on corticospinal excitability expressed as a percentage change for the first and second session (CON group *n* = 10; SON group *n* = 9)

		Mean	Median	SD	SEM	95% CI	
						Lower	Upper
Session 1	SON	36.44	13.62	47.40	14.99	2.53	70.35
	CON	82.41	57.96	82.63	27.54	18.90	145.93
Session 2	SON	5.98	3.89	27.29	8.63	− 13.54	25.50
	CON	12.21	7.48	23.77	7.92	− 6.06	30.49

## Discussion

This study was carried out to investigate the effects of sonification of combined action observation and motor imagery on corticospinal excitability. To this purpose, we trained participants to engage in a practice block comprising congruent AOMI, MI and execution of the same action. The experimental group received sonification during AOMI, while a control group received no sonification. An additional aim of this study was to investigate audiomotor plasticity arising from such training. To do so, we used a variation of an established method to investigate neural plasticity, auditory paired-associative stimulation.

### Combined action observation and motor imagery training and effect of sonification

The primary aim of this project was to investigate the effects of auditory augmentation of AOMI on corticospinal excitability. To this end, participants completed a single practice session based on AOMI, MI and physical execution of the same action. In addition, a SON group received auditory augmentation during AOMI. Sonification yielded no significant facilitation of corticospinal excitability, compared to training without sensory augmentation. Although we are not aware of studies exploring the effects of sonification of corticospinal excitability, neuroimaging and behavioral studies have shown that observing a sonified action induces better movement-related perceptual judgments, a more active engagement of the sensorimotor system during AO (Schmitz et al. 2013), as well as superior performance and rehabilitative outcomes in people with Parkinson’s disease (Mezzarobba et al. 2018). It is possible that sonification did not exert its enriching function during AOMI because the task was straightforward to perform or imagine, rendering the auditory information redundant. There is evidence suggesting that corticospinal excitability is influenced by the vividness of MI (Lebon et al. 2012). Thus, even though the task was straight forward, it may not necessarily mean that it was easy to imagine. However, MIQ results suggest that our participants were, on average, good imagers (c.f. Marchesotti et al. 2016; Vuckovic and Osuagwu 2013), thus further decreasing the value of sensory augmentation. Given the need for accurate coil localization, we were restricted on actions that could be used in this study. Future studies should explore sonification of simulated action using a more ecologically valid action accordingly.

Another possible reason for the lack of effect of sonification on corticospinal excitability may be due to interactions between AO, MI, and external auditory feedback. Recent investigations suggest that combined usage of AO and MI affects attentional processing and mental effort (Bruton et al. 2020; Meers et al. 2020). Studies show that during AOMI, there is a reallocation of attention between externally evoked to internal simulation of the kinesthetic predicted sensation arising from the action (Eaves et al. 2016a, b). Studies investigating corticospinal excitability during various forms of AOMI support this view. Bruton et al. (2020) assessed corticospinal excitability, eye movement and behavioral data while participants engaged in congruent, coordinated, and conflicting AOMI. Congruent AOMI, as used in this study, resulted in significantly higher MEPs and reduced mental effort. Relevant to the present study, however, is the fact that participants reported increased attentional demands during conflicting AOMI, and MEPs were significantly lower than during congruent AOMI. Even though research on sonification suggests that an optimal audiomotor mapping decreases attentional demands and cognitive load of the task (Dyer et al. 2017), and improves performance (Sigrist et al. 2013), there is also evidence suggesting that, compared to other sensory augmentation strategies, sonification may represent an additional attention weight on people, especially early in the training regime (Ronsse et al. 2011). In our study, we used congruent AOMI, which has been shown to require less mental effort, but the addition of sonification may have resulted in comparable increases in attentional demands, thereby negating potential facilitative effects of the former. Further studies are needed to confirm this hypothesis.

Regardless of sonification, however, statistical analysis revealed a practice effect that agrees with the available literature on practice-related neuromodulation. Thus, the training exerted its modulatory effect. Motor learning, with or without sensory augmentation, is characterized by an increase in corticospinal excitability, as measured by TMS (Jung and Ziemann 2009; Rosenkranz et al. 2007a, b; Ziemann et al. 2004). It is thought that the initial phase of learning, the within-session fast learning, is based on an unmasking of silent connections, which are based on LTP-like mechanisms (Pascual-Leone et al. 1995). Studies show that even very simple movements, such as repeated thumb abduction/adduction, produce measurable changes in corticospinal excitability, in line with LTP-like plasticity (Rosenkranz et al. 2007a, b; Ziemann et al. 2004). This mechanistic view of motor learning also applies to more cognitive forms of motor learning, such as AO and MI, as evidence shows that similar plasticity-related modulation of corticospinal excitability are obtained when PAS follows a practice session of observational or mental practice (Avanzino et al. 2015; Lepage et al. 2012). In addition, engaging in AOMI may be better than AO and MI alone (Marshall et al. 2020; Marshall et al. 2019), as it has been linked to increased neural activity (Bruton et al. 2020; Eaves et al. 2016a, b; Wright et al. 2018), thus could potentially influence the rate of practice-dependent plasticity (Eaves et al. 2016). To our knowledge no research has been done on this. Taken together, our results confirm that practicing the pinching of a small object—in this case, a battery—induces an increase in corticospinal excitability of the FDI muscle. The fact that only MEPs, but not the IO curve parameters, exhibited modulation effects suggests that any learning effect was probably small.

In this study, we focussed on sonification of congruent AOMI, which has been the most studied form of AOMI. However, future studies should also explore the effects of sonification of other types of AOMIs such as coordinative and incongruent AOMI (Eaves et al. 2016b; Vogt et al. 2013). Under the dual simulation hypothesis, when the observed and imagined action are not congruent, there is a representational conflict, which results in a lower corticospinal excitability, and an increase in attentional demand to complete the task (Bruton et al. 2020; Meers et al. 2020). However, these forms of dual representation of action can still be used in motor (re)learning and should be further explored. Considering that AOMI implies a change in focus between externally top internally driven action simulation (Eaves et al. 2016a, b; Eaves et al. 2016), sonification could be used to integrate multimodal representation of a complementary aspect of an imagined action. In a hypothetical scenario, a person could imagine performing an action, while simultaneously observing the same action from another point of view and listening to auditory augmentation. Future studies, however, need to further explore whether this hypothesis could have real application to the field of motor (re)learning.

Our discussion regarding the effectiveness, or lack thereof, of sonification for simulation training remains somewhat speculative, given the inconclusive findings. Indeed, the sample size was limited, thus affecting our ability to conclusively discuss the impact of sAOMI for action simulation. Further studies, with a larger sample size, are needed, to further explore this area. Different studies have highlighted the importance of AO and MI for rehabilitation regimes, and its fundamental role in neurological conditions (Abbruzzese et al. 2015; Marshall et al. 2020; Mulder 2007) and immobilization (Bassolino et al. 2014). Under the right conditions, sonification could represent important strategy to maximize learning in clinical conditions, such as stroke survivors (Scholz et al. 2014, 2015, 2016), but could also be a viable sensory substitution strategy for conditions such as deafferentation (Danna and Velay 2017; Danna et al. 2015). Lastly, further development of sonification research may find application in the field of brain-computer interfaces, by affording strategies to improve embodiment of non-body objects, such as neuroprostheses (D’Alonzo et al. 2019; Di Pino et al. 2014a, b, 2020), an issue that crucial for optimal development of the field (Makin et al. 2017).

### The effect of aPAS on corticospinal excitability

On the first visit, we evaluated the effects of aPAS on corticospinal excitability. aPAS produced an increase in MEPs immediately post aPAS, compared to pre-aPAS measures. In addition, for the IO curve parameters resulting from the Boltzmann curve fitting, we observed a significant increase in the maximum evoked potential, as well as a significant shift to the left of the slope of the curve, which is usually interpreted as an increase in corticospinal excitability (Rosenkranz et al. 2007a, b). A significant increase in the range of the evoked potentials is also consistent with the increase in MEP_max_. Our results confirm those of Sowman et al. (2014), who first reported associative LTP-like plasticity within the audiomotor domain by associating a speech sound (the word ‘Hey’) to TMS delivered over the FDI muscle. In our experiment, we used a similar protocol, except that the sound associated to the TMS pulse was a keyboard typing action sound. We used this sound because we stimulated the FDI muscle, which is a prime mover for this action. Our results, however, are very similar to those obtained by Sowman and colleagues. Thus, together with this previous study, our findings suggest that the association of an action sound, regardless of the effector, to a TMS pulse delivered 100 ms after the sound onset at 120% of the individually defined rMT yields a robust modulatory effect on corticospinal excitability.

From a mechanistic point of view, PAS is based on spike-timing-dependent plasticity (STDP). One of the key features of STDP is associativity; that is, its modulating effects are based on the timing of arrival of the two stimuli on the target neuron (Suppa et al. 2017). In most of PAS interventions, an interstimulus interval of 25 ms is usually chosen to induce LTP-like plasticity (Carson and Kennedy 2013; Ranieri et al. 2019; Stefan et al. 2000). We based our protocol on an already published literature on associative plasticity in the audiomotor domain (Sowman et al. 2014). 100 ms from a stimulus onset also coincides with the N100 component of the ERP waveform, which is thought to be related to stimulus-dependent arousal (Naatanen et al. 2011; Nash and Williams 1982). There is evidence that the auditory N100 is influenced by habituation. Indeed, Löfberg and colleagues reported that, when the same auditory stimulation is delivered in trains of four—one per second—corticospinal excitability is increased only for the first stimulus in each train; subsequent TMS pulses yield decreases in corticospinal excitability, suggesting a habituation effect (Löfberg et al. 2018,2014). Nevertheless, we did not find evidence of habituation, as our results confirm that aPAS is a robust technique for modulating corticospinal excitability, consistent with Hebbian learning. In addition, evidence from visuomotor PAS confirms a modulation of corticospinal excitability with an interstimulus interval of 100 and 120 ms (Suppa et al. 2015). Taken together this raise the possibility that the interstimulus interval for cross-modal PAS may be around 100 ms. Further studies are needed, however, to confirm this hypothesis.

### Occlusion of LTP-like plasticity after training

A secondary aim of this study was to gain information on the interaction between sonification and plasticity. To this end, both SON and CON group underwent an additional aPAS protocol after the training session. Evidence suggests that PAS and practice-dependent plasticity share similar neural mechanisms, such that the priming of practice affects the modulatory effects of PAS. Specifically, studies suggest that both motor skill learning and PAS-induced associative plasticity result from a modulation of synaptic strength and weight within the network targeted by the intervention, and this is based on STDP (Caporale and Dan 2008). Evidence also shows that if two LTP-inducing protocol are done in succession, the first protocol interferes with the effect of the second. This form of metaplasticity—plasticity of plasticity—can be induced with two excitatory PAS (Müller-Dahlhaus et al. 2015; Müller et al. 2007) or by priming a PAS with a practice block (Rosenkranz et al. 2007a, b; Stefan et al. 2006; Ziemann et al. 2004).

In our study, both CON and SON performed the same protocol, except for auditory augmentation during AOMI. Considering that in the first session we confirmed the sensitivity of aPAS to audiomotor plasticity, we wanted to explore the interaction between sonification and aPAS, which is designed to test audiomotor connectivity. This could provide evidence of practice-dependent cross-modal interaction. Post-aPAS measures of corticospinal excitability, however, did not report any neuromodulation, compared with post-practice measures for both groups. In addition, for both SON and CON group, the effect of aPAS completed after the practice was lower than the one completed in the first session, and no differences between the groups were found. It is possible that the effect of sonification on learning was small, and that the execution component of the training block masked any effect of sonification. The auditory cortex and M1 do not have direct connections (Cammoun et al. 2015) and, as for the visual processing (Milner and Goodale 2008), auditory processing engages two pathways, a ventral and a dorsal one (Rauschecker and Tian 2000), with the dorsal pathway being responsible for audiomotor integration (Baumann et al. 2007). It is thought that an auditory stimulus engages the motor system via the dorsal route (Rauschecker 2011), which from the thalamus, engages the parietal cortex, where it is integrated with visual and other stimuli (Tanaka and Kirino 2018), to create a multisensory perception (Gottlieb 2007). As highlighted earlier in the text, if a practice block is followed by PAS protocol, an interaction between the two protocols is evident (Rosenkranz 2007a, b; Stefan et al. 2006; Ziemann et al. 2004). MEPs are a motor phenomenon (Hallett 2007; Terao and Ugawa 2002) and, as such, it is possible that the physical execution portion of the training produced a ceiling effect in terms of LTP in M1, so as to mask any effect of sonification on the interaction between practice and aPAS.

Another possible explanation for our aPAS results could be methodological. It is also possible that the temporal spacing between the practice block and subsequent aPAS session influenced participants’ attention levels. There is evidence that the interaction between LTP-like neuromodulatory protocols are sensitive to the spacing between those two protocols (Müller-Dahlhaus et al. 2015). While plasticity arising from motor learning is long-lasting (Dayan and Cohen 2011), the spacing between our two protocols may have affected the level of attention during aPAS. This view is supported by evidence that participants’ level and focus of attention affect the outcome of PAS (Kamke et al. 2012, 2016; Stefan et al. 2004). That is, it is possible that participants may have been unable to sustain high level of attention to the protocol, or worst may have been in a state of drowsiness. However, drowsiness is associated with a decrease in corticospinal excitability (Salih et al. 2005). The fact that after the practice block the effect of aPAS on corticospinal excitability was smaller than the session completed in isolation may be evidence of a suboptimal level of attention to the aPAS stimuli. To mitigate loss of attention, future studies should explore the optimal length of an aPAS protocol, to suggest the minimum number of audiomotor pairing that still neuromodulate corticospinal excitability. It is possible that a shorter aPAS protocol may allow participants to better sustain the practice block and the aPAS protocol. For example a longer break between practice block and post-practice aPAS may be longer, to give participants time to relax, and be more predisposed to the protocol. Lastly, future studies should investigate the neural aftereffects of aPAS to gain evidence on the interaction between the practice block and aPAS, for example with combined TMS-EEG (Hallett et al. 2017; Ilmoniemi and Kičić 2010; Rogasch and Fitzgerald 2013).

To the best of our knowledge, we are the first to explore audiomotor metaplasticity. Our results extend the findings of Sowman et al. (2014), with regard to the effects of aPAS on corticospinal excitability of hand muscles. However, we acknowledge that our study would have benefitted by a larger sample size. Future studies are needed to further explore this protocol. Plasticity is thought to be the underlying neural substrate of learning, and measuring the neuromodulation resulting from the learning process is fundamental for the development of new tools and strategies to maximize learning, and more studies are needed to further elucidate the neuromodulatory effects of aPAS. The development of aPAS may provide an effective test to assess audiomotor connectivity, which may provide, in turn, mechanistic evidence for clinical deficits, as well as the link between the deficit and interventions, via occlusion of LTP-like plasticity (Rosenkranz et al. 2007a, b; Ziemann et al. 2004). Further, aPAS may also represent an intervention tool. Recent studies highlighted the potential therapeutic benefits of using PAS in neurological conditions such as stroke (Silverstein et al. 2019) or incomplete spinal cord injury (Ling et al. 2020); along those lines, aPAS may represent a viable intervention for audiomotor conditions, such as stuttering (Sares et al. 2020). To achieve this, future studies should confirm the optimal ISI. Since PAS is based on STDP (Caporale and Dan 2008), the timing of the arrival of volleys at M1 is crucial.

## Conclusion

In the present study, we investigated the effects of sAOMI on corticospinal excitability, and its neuromodulatory role when paired with aPAS. After a training practice based on sAOMI and physical execution of the action, corticospinal excitability was not modulated, compared to pre-practice measures. In addition, our results confirm previous evidence that aPAS alone modulates corticospinal excitability, evidenced by post-aPAS increases in MEP amplitudes. However, its effects on homeostatic metaplasticity are unclear, and future studies with a larger participant pool may provide more robust evidence of the effects of sonification on action simulation training and audiomotor metaplasticity.

## Supplementary Information

Below is the link to the electronic supplementary material.

Supplementary file1 (DOCX 23 KB)
