# Supplementary material for: Does sonification of action simulation training impact corticospinal excitability and audiomotor plasticity?
Source: Exp Brain Res. 2021 Mar 8;239(5):1489–505. doi: 10.1007/s00221-021-06069-w (PMC8144125; doi:10.1007/s00221-021-06069-w)
Supplement: Supplementary file 1 — Supplementary file1 (DOCX 23 KB) [file 221_2021_6069_MOESM1_ESM.docx]

**S1**: Summary of statistical comparison using rmANOVA on the parameters of the IO curve on the second visit.

| MEP_min_ | | | | | | |
| --- | --- | --- | --- | --- | --- | --- |
| Within Subjects Effects | | | | | | |
| Cases | **Sum of  Squares** | **df** | **Mean  Square** | **F** | **p** | **η²_p_** |
| Time | 2.092 | 2 | 1.046 | 2.407 | 0.105 | 0.124 |
| Time x GROUP | 1.067 | 2 | 0.533 | 1.227 | 0.306 | 0.067 |
| Residuals | 14.778 | 34 | 0.435 |  |  |  |
| Between Subjects Effects | | | | | | |
| GROUP | 0.035 | 1 | 0.035 | 0.029 | 0.867 | 0.002 |
| Residuals | 20.643 | 17 | 1.214 |  |  |  |
|  |  |  |  |  |  |  |
| MEP_max_ | | | | | | |
| Within Subjects Effects | | | | | | |
| Cases | **Sum of  Squares** | **df** | **Mean  Square** | **F** | **p** | **η² _p_** |
| Time | 66.886 | 2 | 33.443 | 1.301 | 0.286 | 0.071 |
| Time x GROUP | 18.214 | 2 | 9.107 | 0.354 | 0.704 | 0.02 |
| Residuals | 874.113 | 34 | 25.709 |  |  |  |
| Between Subjects Effects | | | | | | |
| GROUP | 8.244 | 1 | 8.244 | 0.03 | 0.864 | 0.002 |
| Residuals | 4613.399 | 17 | 271.376 |  |  |  |
|  |  |  |  |  |  |  |
| I_50_ | | | | | | |
| Within Subjects Effects | | | | | | |
| Cases | **Sum of  Squares** | **df** | **Mean  Square** | **F** | **p** | **η²_p_** |
| Time | 32.122 | 2 | 16.061 | 1.096 | 0.346 | 0.061 |
| Time x GROUP | 35.31 | 2 | 17.655 | 1.204 | 0.312 | 0.066 |
| Residuals | 498.461 | 34 | 14.661 |  |  |  |
| Between Subjects Effects | | | | | | |
| GROUP | 37.552 | 1 | 37.552 | 0.476 | 0.5 | 0.027 |
| Residuals | 1340.986 | 17 | 78.882 |  |  |  |
|  |  |  |  |  |  |  |
| Slope | | | | | | |
| Within Subjects Effects * | | | | | | |
| Cases | **Sum of  Squares** | **df** | **Mean  Square** | **F** | **p** | **η²_p_** |
| Time | 38.582 | 1.495 | 25.813 | 3.08 | 0.076 | 0.153 |
| Time x GROUP* | 0.799 | 1.495 | 0.534 | 0.064 | 0.891 | 0.004 |
| Residuals | 212.962 | 25.409 | 8.381 |  |  |  |
| Between Subjects Effects | | | | | | |
| GROUP | 21.274 | 1 | 21.274 | 3.026 | 0.1 | 0.151 |
| Residuals | 119.51 | 17 | 7.03 |  |  |  |
|  |  |  |  |  |  |  |
| RANGE | | | | | | |
| Within Subjects Effects | | | | | | |
| Cases | **Sum of  Squares** | **df** | **Mean  Square** | **F** | **p** | **η²_p_** |
| Time | 48.876 | 2 | 24.438 | 0.886 | 0.422 | 0.05 |
| Time x GROUP | 19.449 | 2 | 9.724 | 0.352 | 0.706 | 0.02 |
| Residuals | 938.145 | 34 | 27.593 |  |  |  |
| Between Subjects Effects | | | | | | |
| GROUP | 7.204 | 1 | 7.204 | 0.026 | 0.874 | 0.002 |
| Residuals | 4726.055 | 17 | 278.003 |  |  |  |
|  |  |  |  |  |  |  |
| SLOPE I_50_ | | | | | | |
| Within Subjects Effects | | | | | | |
| Cases | **Sum of  Squares** | **df** | **Mean  Square** | **F** | **p** | **η²_p_** |
| Time* | 7.642 | 1.013 | 7.543 | 1.582 | 0.226 | 0.085 |
| Time x GROUP* | 2.942 | 1.013 | 2.904 | 0.609 | 0.448 | 0.035 |
| Residuals | 82.095 | 17.222 | 4.767 |  |  |  |
| Between Subjects Effects | | | | | | |
| GROUP | 0.557 | 1 | 0.557 | 0.179 | 0.678 | 0.01 |
| Residuals | 53.043 | 17 | 3.12 |  |  |  |

* Mauchly Test of Sphericity was violated. Greenhouse-Geisser Correction was applied.
